# Mining latent information in PTSD psychometrics with fuzziness for effective diagnoses

**DOI:** 10.1038/s41598-018-34573-7

**Published:** 2018-11-02

**Authors:** Yuanyuan Li, Xi Xiong, Changjian Qiu, Qiang Wang, Jiajun Xu

**Affiliations:** 1 Mental Health Center, West China Hospital of Sichuan University, Chengdu, 610041 China; 2 School of Cybersecurity, Chengdu University of Information Technology, Chengdu, 610225 China

## Abstract

The options of traditional self-report rating-scale, like the PTSD Checklist Civilian (PCL-C) scale, have no clear boundaries which might cause considerable biases and low effectiveness. This research aimed to explore the feasibility of using fuzzy set in the data processing to promote the screening effectiveness of PCL-C in real-life practical settings. The sensitivity, specificity, Youden’s index etc., of PCL-C at different cutoff lines (38, 44 and 50 respectively) were analyzed and compared with those of fuzzy set approach processing. In practice, no matter the cutoff line of the PCL-C was set at 50, 44 or 38, the PCL-C showed good specificity, but failed to exhibit good sensitivity and screening effectiveness. The highest sensitivity was at 65.22%, with Youden’s index being 0.64. After fuzzy processing, the fuzzy-PCL-C’s sensitivity increased to 91.30%, Youden’s index rose to 0.91, having seen marked augmentation. In conclusion, this study indicates that fuzzy set can be used in the data processing of psychiatric scales which have no clear definition standard of the options to improve the effectiveness of the scales.

## Introduction

An earthquake can be a tremendous trauma which might cause the Post-traumatic Stress Disorder (PTSD). The prevalence of PTSD after earthquake, ranges from 4.5% to 95%^1,2^. Although the rate of PTSD can remit with time, it still has great possibility to become chronic and protracted. The prospective studies have shown that the PTSD remission rates range between 35% and 66% after 3–36 months^3,4^ suggesting that there was still a considerable percentage of subjects with PTSD lasting several years and negative impacts on the quality of their lives^4,5^. Meanwhile, PTSD has a high rate of comorbidity with other mental disorders and substance abuses, such as depression^6,7^, specific phobia, contingent anxiety, alcohol misuse^8^, drug abuse^9^ and so on^10^. The delayed treatment may prolong the duration of symptoms of the individuals who do not experience spontaneous recovery^11^. Therefore, confirming the PTSDs among the survivors is crucial and paramount.

There are various scales to screen PTSD. As an earthquake can affect a huge number of people in an immense scale, and thus an easy-to-operate and time-saving screening tool is demanded and called upon. The PTSD Checklist Civilian(PCL-C)^12^ is most widely used among civilians in the PTSD screening. It is a 17-item self-report rating-scale instrument based on the Fourth Edition of the Diagnostic and Statistical Manual of Mental Disorders (DSM-IV; American Psychiatric Association, 1994), taking an average time of 5 to 10 minutes^13^ to complete which requires much shorter time than other scales, such as the Clinician- Administered PTSD Scale^14^ and the PTSD Symptom Scale^15^. As a self-report scale, PCL-C is filled by the subjects themselves without the guide of the interviewers which is much more efficient than the clinician-administered scales. Participants report the extent of their PTSD symptoms on a five-option scale including “not bothered at all” (1), “bothered a little” (2), “bothered moderately” (3), “bothered a lot” (4) and “bothered severely” (5).

In the previous studies the PCL-C has revealed strong validity and reliability^12,16^, while today an increasing number of researchers have pointed out the biases of the traditional scales like the PCL. These traditional methods assume that participants can “fairly accurately” rate their feelings with a definite number. However, several researchers have argued that many psychological constructs are “fuzzy”^17,18^, that is, the definitions and boundaries of these constructs are unlikely to be able to resemble their “hard science” counterparts, such as in explaining physical or biological phenomena. The researchers have also pinpointed that using the traditional crisp scales to directly measure these essentially fuzzy constructs creates an inconsistency that then biases measurement or excludes certain information^17,19,20^. These arguments have been witnessed and supported by psychological and medical studies^21–23^.

In the present study, we still have the same query for the PCL-C, as it is also a traditional questionnaire which doesn’t give clear definitions and boundaries for the options either. The participants cannot fully apprehend or tell the definitions’ differences among the five options- “not bothered at all” (1), “bothered a little” (2), “bothered moderately” (3), “bothered a lot” (4) and “bothered severely” (5). Taking the first item of the scale as an example, “repeated, disturbing memories, thoughts, or images of a stressful experience from the past”, its options are lack of correspondingly substantiated quantitative criteria, such as frequencies, duration, the vividness of the images. Evidently during self-assessment, how could the patients differentiate “bothered a little”, “bothered moderately”, “bothered a lot” and “bothered severely”? If the subjects were having disturbing memories three times a day, should they choose “bothered a lot” or “bothered severely”? Another issue giving concern is that, the same symptoms might be evaluated with different scores by different participants, for example, relatively pessimistic patients tend to choose “bothered severely”, while relatively optimistic patients tend to choose “bothered a little” or “bothered moderately”. Besides, culture, education, personal experience etc. can also affect patients’ proclivity of choosing particular options. Due to the scale options’ vagueness for boundary and patients’ individual differences over the interpretations of the options, the final scores of the PCL-C might be considerably biased and are not possible to reflect the true conditions. Presumably this is partially attributed to the “unstable” presentations of the PCL-C. As the previous studies showed that the sensitivity and specificity of the PCL-C might vary across different populations, therefore cutoff line of the PCL-C have to be changed to ensure the validity and reliability and reduce the biases. While the cutoff line was always chosen by experience or estimate independent of the data itself, could the various cutoff line genuinely reduce the biases of PCL-C, or it may give rise to new biases?

One solution to the basis of traditional scales as proposed by some researchers is to incorporating fuzzy set theory (FST) into the scales^24^. FST is understood as a theory of vagueness or fuzziness and can be used to represent “fuzzy concepts” or vague states of the world. In FST, the membership degrees are normalized as fuzzy values between 0 and 1 rather than crisp values, i.e, 0 or 1. Moreover, consider the difference between fuzzy and crisp information^25^: (a) Tom’s age is 35; (b) Tom is young. The first proposition is crisp (non-fuzzy), namely one can verify its occurrence at the time and map it in terms of a specific numerical measure. The second proposition express “fuzzy information”, namely: what does “young” indicate? How can we represent this concept in order to associate to it a specific numerical measure? To represent the first source of information, we can apply a traditional mathematical framework, but for the second one we can use a specific framework, i.e., FST, which is different than the first one and capable of capturing the main features of fuzzy information.

FST has been applied in various areas such as modeling diagnostic process. Physicians’ expertise was expressed by fuzzy relation of diseases and symptoms^26^, and this approach has been widely used for medical diagnosis. The max-min composition method^27^ and distance-based method^28^ are two popular methods for medical diagnosis based on fuzzy relations^29^. The max-min composition method is intuitive. The distance based method can decrease the loss of data information and assign weights to the symptoms of patients.

FST is a quantitative model that can quantify the uncertainty in human cognition. As a matter of fact, FST has been incorporated into the measurement in psychiatry for decades and has demonstrated its advantages^25^. Most studies first change the forms of the original questionnaires and then use the “new scale” to collect the fuzzy data. According to the previous studies, FST can improve the validity of clinical instruments^30,31^. and reduce the systematic bias in a self-report questionnaire^32^.

The purpose of this study is (1) to investigate the effectiveness of the PCL for screening the PTSD-commission after the earthquake in a local county of China without experimental controls; (2) to explore possible way to incorporate FST into traditional scales such as the PCL and propose a Simplified Fuzzy Option Reduction method called SFOR and (3) to compare the effectiveness of the traditional PCL and the SFOR-processed PCL. We believe that the advantages of SFOR can benefit the PCL studies.

## Methods

### Ethical considerations

This project was approved by the Research Ethics Committee of Sichuan University, the Research Ethics Committee of the West China Hospital of Sichuan University, and the government of Baoxing County and has therefore been performed in accordance with the ethical standards laid down in the 1964 Declaration of Helsinki and its later amendments. Before each data collection session, participants were informed of the aim and procedure of this investigation, and that their participation was voluntary and they had the right to withdraw from the study at any time without penalty. We got the consents from the informed participants before the investigation. For all the adolescent participants, we got the consents from both the participants themselves and their guardians.

### Measures

The general impact of earthquake questionnaire (GIQ) is a brief self-designed questionnaire with only one item- “How severe does the earthquake impact you? ” used to investigate the survivors’ general view of the earthquake. There are five options to indicate corresponding degree of severity for the affected, in ascending order: “not bothered at all” (1), “bothered a little” (2), “bothered moderately” (3), “bothered a lot” (4) and “bothered severely” (5).

The PTSD Checklist, Civilian Version (PCL-C) compiled by Weathers *et al*. in 1993 was used to screen the Post-Traumatic Stress Disorder (PTSD). It is a 17-item self-assessment instrument widely used to measure PTSD-like symptoms identified in the Fourth Edition Diagnostic and Statistical Manual of Mental Disorders (DSM-IV), which constitutes the PTSD diagnosis in the past 30 days, containing three major PTSD symptom clusters: re-experiencing of trauma (Items 1–5), avoidance (Items 6–12), and hyperarousal (Items 13–17). A five-point scale is used for informant responding, with total score obtained by adding the frequency and severity scores across all items (range = 17–85). The cutoff score varies according to the aim of the investigation, the lower the cutoff score is, the higher the sensitivity and the lower specificity are, and vice versa. The recommended cutoff score in previous studies was 44 or 50 which both showed excellent reliability and validity^12^. The cutoff score of 38 was also used in some screening to increase sensitivity and decrease under-diagnosis rate^33^. In this study, we analyzed the data in cutoff scores of 38, 44, and 50, while comparing them with the “fuzzy-processed” result.

The Structured Clinical Interview for DSM-IV Patients (SCID-P) was a clinician-administered diagnostic tool widely accepted as the “gold standard” for the diagnosis of DSM-IV axis-I disorders^34^. Questions were asked exactly as written, and each is based on the individual criteria from DSM-IV. It was translated by the West China Mental Health Institute and revised by the Beijing Huilongguan Hospital Clinical Epidemiological Institute and proved to be with good reliability and validity^35^. All the interviewers were familiar with SCID-P administration and have undergone additional training prior to the survey involving test–retest administration in order to reach a high level of consistency in the administration of the SCID- P. The diagnosis with SCID-P-PTSD took about 20–30 minutes.

### Participants and procedures

**Figure 1:**
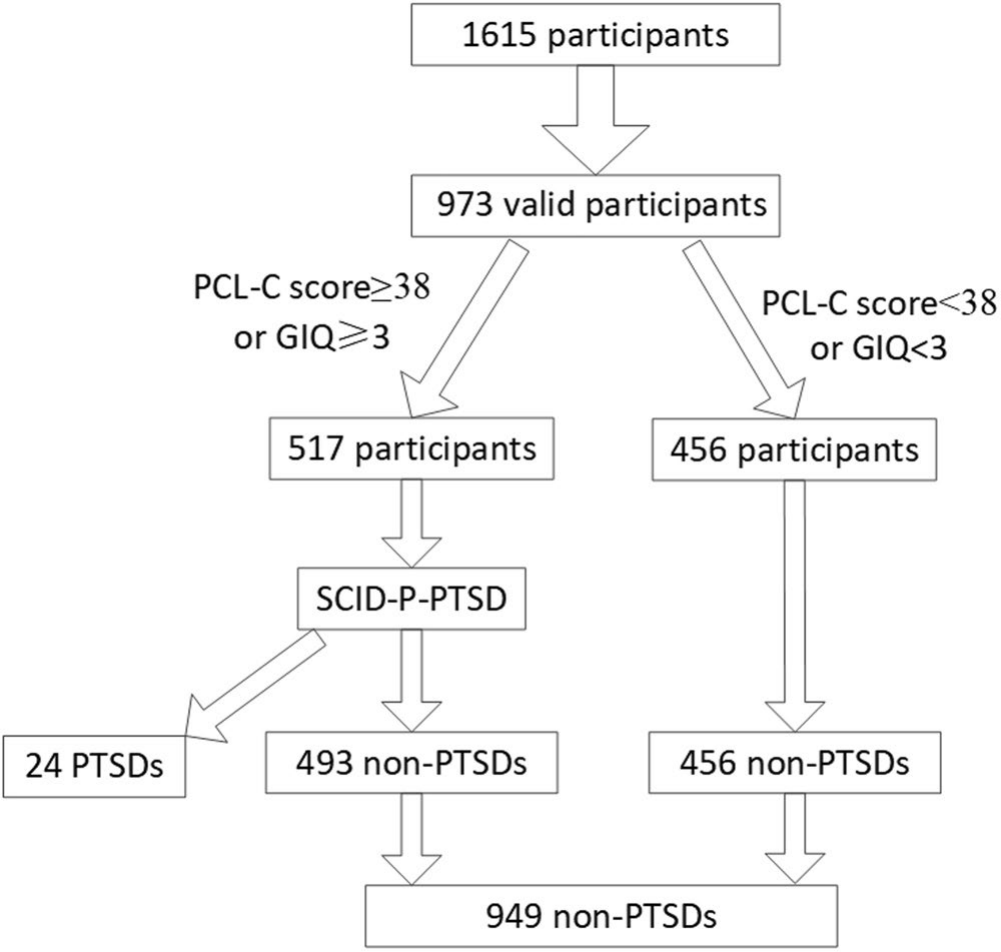
Flow diagram of the sampling strategy.

The present study aimed to investigate the psychological conditions of the people in the disaster-hit region six months after the Baoxing Earthquake, including three steps: 1. assessing demographic variables including age, gender, ethnicity, experience and injuries in the earthquake by using a project-derived questionnaire; 2. evaluating PTSD symptoms by using the PTSD Checklist, Civilian Version (PCL-C); 3. using the Structured Clinical Interview for DSM-IV Patients (SCID-P) to either confirm or exclude PTSD diagnosis in the participants who had a high scores in PCL-C (≥38) and/or believed the earthquake impacted them a lot (GIQ ≥ 3). The SCID-P was given by 4 investigators who had more than ten years’ clinical experience in psychiatry and received training in the use of the diagnostic tools. The consistency of them was between 90% to 100%. If the investigators met an inconsistent diagnosis, they discussed with a professor working in psychiatry department who could help investigators to meet the consistent diagnosis. The sampling strategy is shown in Fig. 1.

### A simplified fuzzy option reduction method (SFOR)

Unlike the conventional set, a fuzzy set expresses the degree to which an element belongs to a set. The characteristic function of a fuzzy set is allowed to have values between 0 and 1, which denotes the degree of membership of an element to a given set.

If *O* is a collection of options denoted generically by *σ*, then a fuzzy option set *A* in *O* is defined as a set of ordered pairs:

1 $$A=\{(x,{\mu }_{A}(x))|x\in U,\,{\mu }_{A}(x)\in [0,1]\}$$

where *μ*_*A*_ is called the membership function for the fuzzy option set. The membership function maps each element of *O* to a membership value between 0 and 1.

Usually *O* is referred to as an option set, and it may consist of limited discrete values. This can be clarified by the following example. If one has several attributes and each attribute can be one of the ten discrete options from 1 to 7. Let Q = {1,4,7} be the set of options one may choose to indicate the option of the attribute. The fuzzy option set A = “the belonging of option 2” may be described as follows:

2 $$A=\{(1,0.7),(4,0.3),(7,0.1)\}$$

Some options are abundant and need to be modified to the adjacent option values which are reserved. If the abundant option is *σ*_*i*_ and its adjacent options are *σ*_*i*1_ and *σ*_*i*2_, for sample *k* (participant *k*), the percentage of attributes with option *σ*_*i*_ that will be modified to option *σ*_*i*1_ and *σ*_*i*2_ is calculated by:

$$\begin{array}{c}{\rho }_{k}({\sigma }_{i}\to {\sigma }_{i1})={{n}_{i1}}^{(k)}/({{n}_{i1}}^{(k)}+{{n}_{i2}}^{(k)})\\ {\rho }_{k}({\sigma }_{i}\to {\sigma }_{i2})={{n}_{i2}}^{(k)}/({{n}_{i2}}^{(k)}+{{n}_{i2}}^{(k)})\end{array}$$

where *n*_*i*1_ and *n*_*i*2_ indicate the number of attributes with option *σ*_*i*1_ and *σ*_*i*2_, respectively. Moreover, we can calculate the number of attributes with option *σ*_*i*_ that will be modified to *σ*_*i*1_ and *σ*_*i*2_ as follows:

3 $$\begin{array}{c}{n}_{k}({\sigma }_{i}\to {\sigma }_{i1})={\rho }_{k}({\sigma }_{i}\to {\sigma }_{i1})\,\ast \,{n}_{k}({\sigma }_{i})\\ {n}_{k}({\sigma }_{i}\to {\sigma }_{i2})={\rho }_{k}({\sigma }_{i}\to {\sigma }_{i2})\,\ast \,{n}_{k}({\sigma }_{i})\end{array}$$

**Figure 2:**
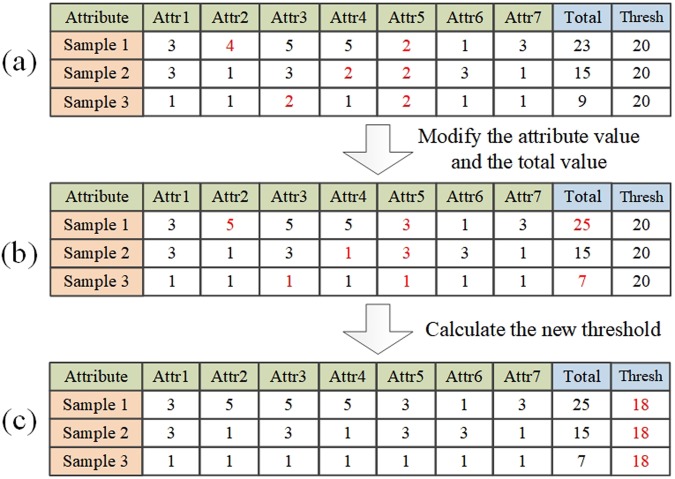
Figure legends should be included at the end of the manuscript file.

Then the diagnosis threshold should be revised accordingly. When option *σ*_*i*_ is modified to *σ*_*i*1_ and *σ*_*i*2_, the degree of threshold variation is computed as:

4 $${{\lambda }}_{i}=\frac{{n}_{i}}{{\sum }_{i}{n}_{i}}\,(\frac{{n}_{i1}}{{n}_{i1}+{n}_{i2}}\,\ast \,\frac{{\sigma }_{i1}}{{\sigma }_{i}}+\frac{{n}_{i2}}{{n}_{i1}+{n}_{i2}}\,\ast \,\frac{{\sigma }_{i2}}{{\sigma }_{i}})$$

where *σ*_*i*1_/*σ*_*i*_ and *σ*_*i*2_/*σ*_*i*_ represents the degree of threshold variation where option *σ*_*i*_ is modified to *σ*_*i*1_ and *σ*_*i*2_, respectively. *n*_*ix*_ represents the number of option *σ*_*ix*_ for all samples instead of one sample. For all the abundant options, the degree of threshold variation is computed as $\lambda ={\sum }_{i}{\lambda }_{i}$, and the new threshold is *th*’ = th*λ. An example is illustrated in Fig. 2.

The cutoff score is calculated according to Equation 4. It takes all samples into consideration and obtain a global value. It mainly depends on the number of each attribute value which is chosen from a specific number of options.

The PCL-C’s data undergone fuzzy processing will be called fuzzy-PLC-C here in after.

## Results

There were 1615 participants recruited from Baoxing County. The inclusion criteria were willingness to participate and age ≥16, and the exclusion criteria were refusal to participate and failure to complete the majority of the investigation. No participants were excluded in the first stage. In the second and third stages 642 participants didn’t finish the investigation. At the end of the data collection, data for 973 participants were obtained for analysis.

### Description of Studied Population

The demographic characteristics are shown in Table 1, reflecting the specifics of gender, age, marital status, ethnicity, experience and injuries in the earthquake. Out of total 973 participants, 627 (62.8%) were female, and 990 (99.2%) were Han. The mean age was 45.81 ± 14.31 years (range: 16 to 81 years). There were 983 (98.5%) adults. 961 (96.3%) of the participants have experienced the earthquake. 59 (5.9%) of them were injured in the earthquake.

**Table 1:** Description of Studied Population (N = 973).

Variables	Value	N (%)
gender	Female/male	614/359 (63.10%/36.90%)
age group	<18/≥ 18	13/960 (1.34%/98.66%)
marital status	married/single	855/118 (87.87%/12.13%)
ethnicity	Han/minority	895/78 (91.98%/8.02%)
having experienced the earthquake	yes/no	948/25 (97.43%/2.57%)
injured in the earthquake	yes/no	50/923 (5.14%/94.86%)

### The Cross Tabulation of GIQ and the PCL-C

Table 2 is the crosstab of the PCL and GIQ. The number of participants with the PCL ≥ 38 was 105. GIQ ≥ 3 meant the earthquake bothered the individual at least moderately. The number of participants with GIQ ≥ 3 was 511. The total number of participants with either the GIQ ≥ 3 or the score of PCL-C ≥ 38 was 517. Finall, 517 individuals received the SCID-P-PTSD.

**Table 2:** The crosstab of the PCL-C and GIQ.

	The score of PCL-C		Total
	≥38	<38	
The score of GIQ ≥ 3	99	412	511
The score of GIQ < 3	6	456	462
Total	105	868	973

GIQ ≥ 3 means the earthquake has bothered the individual at least moderately.

### The diagnosis values of the PCL-C at different cutoff scores and fuzzy-PCL-C

Table 3 shows the diagnosis values of PCL-C at different cutoff scores and the fuzzy-PCL-C. Statistical analysis and calculation were performed by using SPSS (v19.0) with two-tailed p values < 0.05, being considered as statistically significant. Continuous variables were represented by mean value and standard variance. Sensitivity, specificity and Youden’s index were used to evaluate the potency of the PCL-C and fuzzy-PCL-C. Youden’s index is a function reflecting on sensitivity and specificity calculated by adding sensitivity with specificity minus 1. The index ranges from 0 to 1 with the higher values designating elevated effectiveness^36^.

**Table 3:** The diagnosis values of PCL-C at different cutoff scores and the fuzzy-PCL-C.

	PCL-C Cutoff at 50	PCL-C Cutoff at 44	PCL-C Cutoff at 38	fuzzy-PCL-C Cutoff at 27
True positive	7	10	15	22
False positive	42	58	90	138
True negative	907	891	859	836
False negative	17	14	9	2
sensitivity	30.43%	43.4%	65.22%	91.30%
specificity	98.26%	98.56%	99.08%	99.76%
Positive predictive value	14.29%	14.71%	14.29%	13.75%
Negative predictive value	95.57%	93.89%	90.52%	88.09%
Youden’s index	0.29	0.42	0.64	0.91

Sensitivity = true positives/(true positives + false negatives). Specificity = true negatives/(true negatives + false positives). Positive predictive value = true positives/(true positives + false positives). Negative predictive value = true negatives/(true negatives + false negatives). Youden’s index = Sensitivity + Specificity-1.

All the 517 individuals who were either with GIQ ≥ 3 or with score on the PCL-C ≥ 38 received the face to face interview by psychiatrists using SCID-P-PTSD. In the end, the diagnosis of PTSD was given to 24 individuals. The prevalence of PTSD in this sample was 2.47%.

When the cutoff line of the PCL-C was set at 50, 49 individuals scored above the cutoff line where 7 were true positive and 42 were false positive. Among the 924 individuals with scores below 50, 907 were true negative and 17 were false negative. When the cutoff line of the PCL-C was set at 50, the sensitivity was 30.34%, and the specificity was 98.26%, while the positive predictive value was 14.28%, and the negative predictive value was 95.57%, with Youden’s index to be 0.29.

When the cutoff line of the PCL-C was set at 44, 68 individuals scored above the cutoff line where 10 were true positive and 58 were false positive. Among the 905 individuals with scores below 44, 891 were true negative and 14 were false negative. When the cutoff line of the PCL-C was set at 44, the sensitivity was 43.4%, and the specificity was 98.56%, while the positive predictive value was 14.71%, and the negative predictive value was 93.89%, with Youden’s index to be 0.42.

When the cutoff line of the PCL-C was set at 38, 105 individuals scored above the cutoff line where 15 were true positive and 90 were false positive. Among the 868 individuals with scores below 38, 859 were true negative and 9 were false negative. When the cutoff line of the PCL-C was set at 38, the sensitivity was 65.22%, and the specificity was 99.08%, while the positive predictive value was 14.29%, and the negative predictive value was 90.52%, with Youden’s index to be 0.42.

After the SFOR processing, the cutoff score was revised to be 27 according to Equation 4 (as indicated on that the cutoff line of fuzzy-PCL-C was equal to 27). There were 150 individuals above the cutoff line, with 22 of them were true positive, and 138 were false positive. Among the individuals below the cutoff line, there were only 2 false negative, and 836 true negative. The sensitivity of fuzzy-PCL-C was 91.30%, the specificity was 99.76%, while the positive predictive value was 13.21%, the negative predictive value was 88.09%, with Youden’s index to be 0.91.

## Discussion

PCL-C is a self-report scale to be used without the need for professional guidance. It is time-saving and easy-to-operate which is particularly useful to screen PTSD for major catastrophic events (such as earthquake, tsunami and cyclone) affected region’s population, where the area and number of people being impacted are tremendously large.

This study measured the screening effectiveness of the PCL-C at various cutoff lines, six months after Baoxing Earthquake, discovering the good specificity of the PCL (all above 98%), but with less satisfactory sensitivity- even when the cutoff line was set below 38, the sensitivity merely increased to 65.22%, with maximum Yonden’s index to be 0.64. And thus as a screening scale, the PCL entails considerable false negatives rendering its relatively poor screening effectiveness. From our research, it was found that the subjects universally rated (evaluated) their symptoms below than “it-should-be”- when the cutoff line was set at 50, only 49 subjects scored above the cutoff line, accounting for 5%; even when the cutoff line was set at 38, no more than 105 subjects scored above the cutoff line, or namely only 10% subjects scored above the cutoff line.

After examining, we thought the main factors contributing to this result are: the boundaries of the scale’s options are blurry, leading to subjects’ discrepancies in understanding, giving rise to skewedness. Also, the options of the PCL are all of “extent rating” but with nil objective definition on each. As a result, the subjects tend to reply based on their own experience that can be subjective. Some researchers suggest that most people will likely to show comparative optimism- the belief that their personal susceptibility to negative events was less than that of the average person^23,37^. Considering this research was carried out six months after the earthquake, when compared with if it being carried out immediately after the earthquake, survivors’ lives have been back in order, status being restored, so conceivably they showed comparative optimism. Aforementioned factors consequently yield lower score assuming decreased sensitivity of the PCL-C.

According to previous studies, the PCL have good sensitivity and specificity, which is contrary to what we have found in this research. However, it is noted that: in previous researches pertaining the sensitivity and specificity of the PCL, experimental conditions were relatively well controlled, e.g., 1. the homogeneity of the population is fairly good. As the PCL per se has three versions- PCL-C, PCL-M, and PCL-S for measuring their respective population. Also in practice, subjects are normally required to be in comparable profession, age range, and educational level^38^; 2. sampling areas were fairly concentrated, such as in the same community, village etc.; 3. the sample size was not very large, mostly below 500^39^. With these controls of experimental conditions, it to some degree had decreased the effect of individual differences on subjective discrepancies among the people in some specific populations, e.g., people with the same profession or residing in the same area for a long time might have more similar evaluation for the extent of certain symptoms, more likely to score in a uniform manner. Besides, these research results can only prove the PCL’s good sensitivity and specificity within the sampled population, with little to none comparable diagnostic effectiveness can be extrapolated to other samples. In our research, we did not exercise experimental control for subjects, but incorporating multiclass and diversified sampling, which might in turn constitute one of the reasons leading to decreased sensitivity of the PCL. In reality, the screening effectiveness of the PCL is always unstable. Studies regarding the psychometric properties of the PCL has been continuously ongoing^12,38,39^. In practical setting, researchers vary the cutoff lines to improve the screening effectiveness of the PCL^12,40^, some researches even adjust the cutoff line down to 26, for better sensitivity^41^.

Although varying the cutoff line could improve the scale’s sensitivity, when using this method in the process of actual application, it poses problems: 1. altering the cutoff line merely changes the boundaries globally, neglecting the effect of individuals’ different understandings on the scale’s options, whereas the very main reason behind skewed results indeed lies on the discrepancies of individuals’ subjective judgment, therefore altering the cutoff lines may not be able to essentially improve the diagnostic effectiveness of the PCL; 2. the choice of cutoff line normally relies on experience or estimate, not chosen based on the characteristics of data, e.g. in most literatures, the cutoff lines of 50 or 44 are chosen, however whether this choice is suitable for the specific population is not clear^42^. In our research, it is notably witnessed that the sensitivity of the PCL is not quite good, when the cutoff line is at 50, 44, or 38.

To our knowledge, the present study utilizing a fuzzy method to help psychiatrists make decisions provides new perspectives and thoughts to rectify biases, or namely, eliminating unnecessary options, spacing out options, reducing “noise”, making the meaning of each option clearer. Meanwhile, the cutoff lines were adjusted according to the changes of options, simultaneously preserving the scale’s diagnostic principles. After the fuzzy processing, Youden’s index rose from 0.29 to 0.91. Youden’s index is a single statistic that captures the performance of a dichotomous diagnostic test. Youden’s index is calculated by adding sensitivity and specificity then minus 1, meaning that the larger is the Youden’s index, the better is the screening effectiveness. Fuzzy processing maintained the previously good specificity of the scale, and increased sensitivity to 91.30%, then markedly elevated Youden’s index. As a screening scale, the higher is the sensitivity, the more capable it can detect patients. Fuzzy processing heightened the screening effectiveness of the scale, meanwhile preserved its good specificity, and optimized the diagnostic effectiveness of the scale.

Comparing with traditional methods, the advantages of fuzzy processing on data are not only limited to increasing the scale’s effectiveness, but also benefit the front line clinicians in the following ways: 1. The approach that traditional methods used to augment screening effectiveness is by changing cutoff lines, however this approach is merely regulating on global level, being equivalent to inclusion of more samples, whereas the biases are not removed; while fuzzy processing on data does “process” every individual, eliminating or controlling the subjective biases on individual level, making results better reflect patients’ true conditions. As fuzzy processing reduces individual differences- the effects of different cultural backgrounds, personalities, educations, past experiences etc. are lessened. As a result, there is less experimental control efforts required to enhance screening efficiency, having significantly streamlined sample collecting procedures. Furthermore, fuzzy processing is highly appropriate to be applied for samples with complex demographic composition, such as for survivors of an earthquake, in which the disaster area, impacted scale, and affected population are tremendously large; 2. The cutoff line choosing of fuzzy processing is not based on experience, but based on the properties of the data per se, therefore it is more precise, making omission due to improper choosing of cutoff line become less likely.

In summary, the present study has affirmed that fuzzy processing is an efficient and convenient data treatment method in clinical setting. However, it needs to be further verified to authenticate feasibility and stability in future clinical practice. For most clinicians, they might not be familiar with how to make use of fuzzy processing. Upon the respective algorithms being further validated, related software can be programed for assisting clinicians’ diagnosis. In addition, the compilation of the PCL-C scale used in this study is based on DSM-IV. Following the publication of DSM-5, the PCL has been accordingly updated to PTSD Checklist for DSM-5 (PCL-5). The PCL-5 is a 20-item self-report measure that assesses the 20 DSM-5 symptoms of PTSD. The self-report rating scale is 0–4 for each symptom, reflecting a change from 1–5 in the DSM-IV version. Rating scale descriptors are the same: “Not at all,” “A little bit,” Moderately,” “Quite a bit,” and “Extremely”. PCL-5 has only one version which is most similar to the PCL-S (specific) version. There are no corresponding PCL-M or PCL-C versions of PCL-5. Currently PCL-5 has not been widely used in clinical practice, still awaiting considerable researches to verify its sensitivity, specificity, and cutoff lines designation [9, 10]. From a structural point of view, PCL-5 is identical to PCL. Our research has broken through the traditional PCL’s scoring mode, which might provide new insights for the analysis and application of future’s PCL-5. Therefore, further studies are expected.

Some flaws existing in the proposed SFOR method are listed as follows:

1. Before SFOR processed the samples, missing data should be removed, which results in the loss of information. Some filling method such as collaborative filtering^43^, can suggest the missing values from other similar samples with integrated data when the missing data accounts for a small percentage.
2. The choosing of reserved options is based on our experience and the options’ meaning. It can reserve the “valuable” options and remove the “fuzzy” options, but the process seems subjective. How to choose the reserved options by analyzing the inherent features and the mutual relationship of initial data is the area to be studied in the future.
